# When to Take Medicine

**Published:** 1883-10

**Authors:** 


### WHEN TO TAKE MEDICINE.

THE chief causes of disease are ERRORS IN DIET, ERRORS IN DRESS, INTEMPERANCE, IMPURE WATER, UNWHOLESOME FOOD, DEFECTIVE TEETH, and BLOOD POISONING from inhalation of IMPURE AIR and NOXIOUS GASES. Disease induced by any one of these causes, almost invariably manifests itself by disorder in the functions of the LIVER ; there the alarm is sounded first, and if not attended to promptly the trouble is liable to extend to other vital organs. DYSPEPSIA, CONSTIPATION, CHRONIC DIARRHŒA, DISEASE OF THE KIDNEYS, DROPSY, RHEUMATISM, CATARRH, CONSUMPTION and various forms of SKIN DISEASES often proceed directly from derangement of the LIVER. Attention to the liver forestalls other diseases. We have, fortunately, a few remedies that seem to have a specific action in this direction ; but it is important to select one that acts promptly and thoroughly in order to lessen the danger to other organs through delay. In his book " Health at Home" Dr. Hall says that in a medical practice of twenty years in the lowlands of Louisiana, where hardly a person escapes FEVER AND AGUE, he invariably prescribed his OLD TIME LIVER PILLS, and that they never failed to cure any case that was curable. He gave these pills for all diseases that seemed to emanate from torpid liver, which was in effect saying that he prescribed them in almost all diseases. He especially advises his patients to use them.

WHEN a person residing in a malarial district begins to feel some of the well-known symptoms of approaching CHILLS AND FEVER, as fatigue, head-ache, back-ache, aching of the joints, sleepiness and an inclination to stretch out the limbs, take 4 to 6 pills on going to bed. Remain in the house two or three days and eat sparingly of plain foods. Repeat the dose of pills on the seventh night. In nine cases out of ten no other treatment will be required.

WHEN you feel restless, melancholy and uncomfortable, with perhaps a dull head-ache, there is a torpid liver. Take 3 to 6 pills on going to bed, and go about your business the next day ; but regulate your diet so that there will be no return of the trouble.

WHEN you have taken a sudden cold take 2 to 4 pills on going to bed. Attend to your diet and avoid taking cold again. You will have no more trouble.

WHEN you have an attack of rheumatism, it is due to an unhealty condition of the secretions. Start the liver with 3 or 4 pills taken at bedtime, and repeat the dose in three days if necessary.

To obtain the greatest benefit from any medicine care should be observed as to diet, and whatever else is essential to the promotion of health. These pills have a wonderfully beneficial effect through their action on the LIVER, but no medicine should be depended on exclusively. Medicine is only a factor in the work of curing disease.

DR. HALL'S " OLD TIME " LIVER PILLS are sugar-coated and put up in handsome paper boxes with sliding covers. There is an excellent likeness of Dr. W. W. Hall on each box, and the formula from which the pills are made is printed on the cover. These boxes are larger than the ordinary boxes and contain more pills, but they are sold at the usual, price, 25 cents a box—five boxes for $1.00. Sent postpaid on receipt of price. These pills are manufactured by Hall &Ruckel, wholesale druggists, New York. But for thirty years people have been sending to us for them, and we will not now refuse their orders. Address HALL'S JOURNAL OF HEALTH, New York

